# Periscope upon the Passage of the White Blood Corpuscles through Capillary Walls

**Published:** 1870-07

**Authors:** 


### Periscope upon the Passage of the White Blood Corpuscles through Capillary Walls.

In the *Comptes Rendus,* Jan. 17, M. Robin criticises a work by M. Feltx. His remarks are translated in the *Bowdoin Scientific Review*:

The work of M. Feltz treats of the passage of the leucocytes, or white globules of the blood, through the vascular walls. M. Feltz at the outset briefly recapitulates the theory of Cohnheim, which is itself based upon the anatomical aud histological data furnished, chiefly, by Recklinghausen. Cohnheim does not doubt the passage of the white globules through the *stomata,* of which he assumes the existence in the vessel-walls in case of inflamation, etc.

M. Feltz combats the views of Cohnheim.

1st. He has studied experimentally the circulation in the Mesentery and tongue of the frog, and on the mesentery of the mouse. He concludes that there is primarily a contraction of the vessels, followed by dilation due to temporary loss of the contractility of the vascular walls. Notwithstanding numerous and minute observations, he has never been able (in opposition to Cohnheim) to discover any white globules entering the canaliculi, the existence of which has been admitted through the walls. He has only seen accumulations of leucocytes along their outer and inner walls; but it is impossible for him to affirm, as the result of direct observation, that the extra-vascular globules are an immediate blood-product.

2d. The author has then demonstrated, by different means (injections of colored substances into the blood and lymphatic systems, colorations of the arterial and venous tissues by nitrate of silver, microscopic preparations with photographer's paper, etc.,) that the passage of the leucocytes is impossible, because the pretended epithelial stomata, and the canaliculi leading from them, do not exist; or at least are not shown through the agencies employed by Cohnheim and Recklinghausen.

3d. M. Feltz does not deny that there is around the vessels, in the inflamed tissues, a great number of elements similar to leucocytes; but not having seen them pass out of the vessels, nor formed within pre-existing elements, as the theory of Virchow would establish, he demands whether these elements be not formed *in situ* within exuded fluids. Without advancing a theory regarding their generation, he has entered upon a course of new experiments, which he will soon have the honor of submitting to the Academy.—*Philadelphia Reporter.*

